# 

**Published:** 1855-04

**Authors:** 


### CONTENTS OF THE APRIL NUMBER.

### ORIGINAL COMMUNICATIONS.

Page.

ART. I.—Iodide of Iron. By Richard W. Nelson, M. D. A paper read before the Buffalo Medical Association at its meeting for February, 1855, - - - - - - - - - - 642
ART. II.—Theories of the Production of Males and Females. By Silas Hubbard, M. D., - - 645
ART. III.—The Liver, viewed as a Sugar-producer. A Thesis, presented to the Faculty of the University of Buffalo. By William Howell, - - - - - - - - - - - - 654
ART. IV.—Case of Rupture of the Uterus. By C. Milford Crandall, M. D., Belfast, N. Y., - - 663
ART. V.—A Practical Treatise on Foreign Bodies in the Air Passages. By S. D. Gross, M. D., - 664
ART. VI.—An Inquiry into the Pathological Importance of Ulceration of the Os Uteri, - - 665
ART. VII.—What to Observe at the Bed-side and after Death in Medical Cases, - - - - 667
ART. VIII.—A Manual of Pathological Anatomy, - - - - - - - - - - - 668
ART. IX.—The Principles and Practice of Obstetric Medicine and Surgery, in reference to the Process of Parturition, - - - - - - - - - - - - - - - - - - 668
ART. X.—Table of Meteorological Conditions in the City of Buffalo for February, 1855. By Sanford B. Hunt, M. D., - - - - - - - - - - - - - - - - - 669
ART. XI.—Monthly Record of Deaths in the City of Buffalo. By J. Root, Health Physician, M. D., 670

### ECLECTIC DEPARTMENT.

Simaba Cedron in Intermittent Fever, - - - - - - - - - - - - - - 672
Croup, - - - - - - - - - - - - - - - - - - - - - - 673
On the Operative Method for the Permanent Cure of Aernia, - - - - - - - - 675
Report on Carbuncle, - - - - - - - - - - - - - - - - - 679
New Mode of Opening an Ovarian Cyst, - - - - - - - - - - - - - 680
Ulceration of the Legs, - - - - - - - - - - - - - - - - - 681
On Fracture of the Skull, - - - - - - - - - - - - - - - - 686
Introduction of a stick or swab-handle more than ten inches long into the stomach, - - - 689
Surgery of the War, - - - - - - - - - - - - - - - - - - 691

### EDITORIAL DEPARTMENT.

Pamphlets Received, - - - - - - - - - - - - - - - - - 693
On Injecton of the Bronchial Tubes, and Tubercular Cavities of the Lungs. - - - 693
Anniversary Discourse before the New York Academy of Medicine, - - - - - 695
Oration delivered before the Physico-Medical Society of New Orleans, - - - - - 696
The Transactions of the New Hampshire Medical Society, - - - - - - - - 696
Clark on Puerperal Peritonitis, - - - - - - - - - - - - - - - 697
Knapp on the "Cause, Nature, Cure, and Prevention of Epidemic Cholera," - - - - - 698
Remarks on Granular Conjunctiva, - - - - - - - - - - - - - - 700
Dr. Cammann's Stethoscope, - - - - - - - - - - - - - - - - 701
Case of Vertebral Dislocation, - - - - - - - - - - - - - - - 702
Correction, - - - - - - - - - - - - - - - - - - - - - 703

